# Recent Advances in Traffic Sign Recognition: Approaches and Datasets

**DOI:** 10.3390/s23104674

**Published:** 2023-05-11

**Authors:** Xin Roy Lim, Chin Poo Lee, Kian Ming Lim, Thian Song Ong, Ali Alqahtani, Mohammed Ali

**Affiliations:** 1 Faculty of Information Science and Technology, Multimedia University, Melaka 75450, Malaysia; 2 Department of Computer Science, King Khalid University, Abha 61421, Saudi Arabia; 3 Center for Artificial Intelligence (CAI), King Khalid University, Abha 61421, Saudi Arabia

**Keywords:** traffic sign recognition, machine learning, deep learning

## Abstract

Autonomous vehicles have become a topic of interest in recent times due to the rapid advancement of automobile and computer vision technology. The ability of autonomous vehicles to drive safely and efficiently relies heavily on their ability to accurately recognize traffic signs. This makes traffic sign recognition a critical component of autonomous driving systems. To address this challenge, researchers have been exploring various approaches to traffic sign recognition, including machine learning and deep learning. Despite these efforts, the variability of traffic signs across different geographical regions, complex background scenes, and changes in illumination still poses significant challenges to the development of reliable traffic sign recognition systems. This paper provides a comprehensive overview of the latest advancements in the field of traffic sign recognition, covering various key areas, including preprocessing techniques, feature extraction methods, classification techniques, datasets, and performance evaluation. The paper also delves into the commonly used traffic sign recognition datasets and their associated challenges. Additionally, this paper sheds light on the limitations and future research prospects of traffic sign recognition.

## 1. Introduction

The purpose of this study is to delve into the latest developments in traffic sign recognition using deep learning techniques. As the demand for autonomous vehicles continues to grow, the reliability of traffic sign recognition algorithms is becoming increasingly important for ensuring the safety of all road users.

Traffic sign recognition technology enables vehicles to read and understand important road signs, such as speed limit signs, danger signs, and turn ahead signs. This technology not only improves the safety of drivers, but also contributes to a safer road environment for all users by providing essential information and reminders of important regulations.

In order to fully comprehend the state-of-the-art work in this field, this study conducts a comprehensive review of existing work on traffic sign recognition. These studies are divided into two categories: conventional machine learning and deep learning approaches. Additionally, this study explores the commonly used traffic sign recognition datasets and their associated challenges as well as the limitations of current studies and future research prospects. The findings of this study provide valuable insights for researchers and practitioners working in the field of autonomous vehicle technology.

The main contributions of this paper are as follows:

- A comprehensive review of state-of-the-art traffic sign recognition work, categorizing studies into conventional machine learning and deep learning approaches.
- A discussion of widely adopted traffic sign recognition datasets, their challenges, and limitations, as well as the future research prospects in this field.

The paper is organized into several sections to provide a clear overview of the research. Section 2 presents a brief explanation of research on traffic sign recognition algorithms. In Section 3, existing machine learning methods for traffic sign recognition are reviewed, while Section 4 covers existing deep learning methods. Section 5 discusses widely used datasets in the traffic sign recognition field. The limitations of existing work on traffic sign recognition are presented in Section 6. In Section 7, potential areas for future research on traffic sign recognition are highlighted. Finally, Section 8 provides a conclusion for the paper.

## 2. Traffic Sign Recognition Algorithms

In this section, we review the existing research in the field of traffic sign recognition. The process of traffic sign recognition involves several key steps, including data preprocessing, feature extraction, and classification.

**Figure 1:**
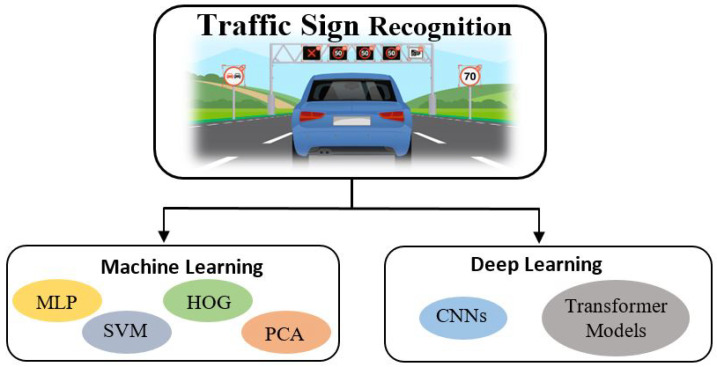
Traffic sign recognition can be divided into machine learning and deep learning approaches.

As shown in Figure 1, the classification algorithms used in traffic sign recognition can be broadly divided into two categories: machine learning and deep learning. This section covers the different studies that have been carried out in these two categories.

Machine learning algorithms include traditional methods, such as Support Vector Machines (SVMs), k-Nearest Neighbor (k-NN), and decision trees. These algorithms are based on the concept of training a model on a dataset and then using that model to make predictions on new data. They have been widely used in traffic sign recognition due to their simplicity and efficiency.

On the other hand, deep learning algorithms use neural networks to model complex relationships between inputs and outputs. They have recently gained popularity in traffic sign recognition due to their ability to automatically learn high-level features from raw data, reducing the need for manual feature extraction. Deep learning algorithms, such as Convolutional Neural Networks (CNNs) and Recurrent Neural Networks (RNNs), have been applied to the problem of traffic sign recognition with promising results.

The following sections provide a detailed overview of existing work on traffic sign recognition. This provides the reader witha comprehensive understanding of the state-of-the-art work conducted in this field and lays the foundation for future research. A comparison of machine learning and deep learning approaches is presented in Table 1.

For the classification of traffic signs, a comparison between machine learning models and deep learning models was made.

## 3. Machine Learning for Traffic Sign Recognition

The field of traffic sign recognition has seen a significant amount of research in recent years, particularly regarding the use of machine learning techniques to classify traffic signs accurately. In a study by Kerim and Efe (2021) [1], an Artificial Neural Network (ANN) was developed to incorporate various features, including Histograms of Oriented Gradients (HOG) and a combination of color, HOG, and Local Binary Patterns (LBP). This hybrid ANN was made up of 9 individual ANNs, each responsible for analyzing traffic signs based on a set of attributes present in the images. The authors used data augmentation techniques such as translation, rotation, and noising to improve the performance of the model. The results showed that the method combining color, HOG, and LBP features achieved an accuracy level of 95%, significantly outperforming the method using HOG features alone.

Another study by Soni et al., (2019) [2] used HOG and LBP descriptors with the Principal Component Analysis (PCA) and Support Vector Machines (SVM) to classify traffic signs. The study used the Chinese Traffic Sign Database (TSRD) with 58 classes and 6164 images, and the best performing method was the LBP with the PCA and SVM classifiers, achieving an accuracy level of 84.44%.

In Namyang and Phimoltares (2020) [3], a combination of the Support Vector Machines (SVM) and Random Forest algorithms was used with HOG and the Color Layout Descriptor (CLD) to classify traffic signs. The authors collected a dataset of 408 training images and 216 testing images, consisting of 4 classes of traffic signs, namely regulatory, warning, construction, and guide signs. The images were first preprocessed to resize them to 120 × 80 pixels. The first stage of the method used HOG features with SVM and a radial basis function (RBF) kernel to classify regulatory signs. The construction class was then classified with SVM, while the warning and guidance signs were classified in the next stage using a hierarchical classification model with HOG and CLD. The method achieved an accuracy level of 93.98%.

Li et al., (2022) [4] presented an approach for traffic sign recognition with finely crafted features and dimension reduction. The authors utilized the color information of traffic signs and enhanced the discrimination between images using the improved color-histogram-based feature. Subsequently, the PCA algorithm was adopted to reduce the dimensions of the improved color-histogram-based feature, which increased the running speed of the method. Lastly, the expression ability of features was further enhanced by concatenating the improved color-histogram-based feature after dimensionality reduction with the HOG feature of images. The experimental results recorded an accuracy level of 99.99% on the German Traffic Sign Recognition Benchmark (GTSRB) dataset.

The paper by Madani and Yusof (2018) [5] presented a traffic sign recognition technique based on three key components: border color, shape, and pictogram information. The proposed technique consists of three independent stages: Firstly, the border colors are extracted using an adaptive image segmentation technique based on learning vector quantization. Secondly, the shape of the traffic sign is detected using a fast and simple matching technique based on the logical exclusive OR operator. Lastly, the pictogram is extracted and classified using a SVM classifier model. The proposed technique was tested on the German traffic sign recognition benchmark, achieving an overall recognition rate of 98.23%.

Sapijaszko et al., (2019) [6] proposed a traffic sign recognition system that comprises normalization, feature extraction, compression, and classification stages. The images are normalized using gray scaling and anisotropic diffusion techniques. The discrete wavelet transform and discrete cosine transform extract essential features from the images while reducing their size. Finally, a three-layer feed-forward multilayer perceptron is used for analysis and classification. The best algorithms achieved a recognition accuracy of 96.0% on the Belgian Traffic Sign dataset (BTSD), 95.7% on the GTSRB, and 94.9% on the TSRD.

Aziz and Youssef (2018) [7] proposed a traffic sign recognition system that leverages feature extraction and the Extreme Learning Machine (ELM) algorithm. The authors evaluated three feature extraction techniques, namely HOG, Compound Local Binary Patterns (CLBP), and Gabor features, and passed the extracted features into ELM for classification. ELM operates on the assumption that learning models can be fed by randomly selected input weights without requiring specific distribution adjustment. The authors tested their proposed method on two datasets, the GTSRB and the BTSD, and achieved high accuracy rates of 99.10% and 98.30%, respectively.

Weng and Chiu (2018) [8] presented a traffic sign recognition that was divided into two stages. In the detection stage, potential traffic signs were detected using the Normalized RGB color transform and Single-Pass Connected Component Labeling (CCL). In the second stage, HOG was used to generate the descriptors of the signs, which were then classified using the SVM. The proposed method achieved a recognition rate of 90.85% when tested with the GTSDB dataset.

Wang (2022) [9] proposed a traffic sign classification system using three machine learning classifiers: Logistic Regression (LR), Multilayer Perceptron (MLP), and SVM. The authors used the Multinomial Logistic Regression classifier, which is a variation of LR that generates a probability distribution indicating the likelihood of each class. They applied the Softmax function to transfer the weighted sum of characteristics into a probability distribution. For MLP, the authors used a biological neuron model to determine its structure and the activation function. For SVM, the authors used the one-vs.-the-rest method with the LinearSVC algorithm. The authors conducted experiments on the GTSRB dataset and achieved accuracy rates of 97.75% for LR, 98.88% for MLP, and 95.51% for SVM. A summary of machine learning methods for traffic sign recognition is provided in Table 2.

## 4. Deep Learning for Traffic Sign Recognition

The field of traffic sign recognition has seen numerous advances in recent years, with many researchers turning to deep learning techniques to develop efficient and accurate algorithms. In this section, we review some of the most notable works in this area.

Siniosoglou et al., (2021) [10] proposed a deep autoencoder algorithm for detecting and recognizing traffic signs. The autoencoder was designed to generate efficient coding for unsupervised learning, with a number of techniques employed to improve its performance, including the use of ReLU activation, upsampling, and convolution strides. The model was trained using the Carla Traffic Signs Recognition Dataset (CATERED), which contained 94,478 images of traffic signs from 43 different classes. The proposed method was tested in two scenarios: a centralized detection system and a decentralized system. In both cases, the method achieved an accuracy of over 90%, with the highest accuracy of 99.19% obtained from the centralized detection system and an accuracy of 94.19% obtianed from the decentralized system.

In another study, Li and Wang (2018) [11] used a Convolutional Neural Network (CNN) with a pretrained model, MobileNet, for traffic sign recognition. The MobileNet architecture was designed to be lightweight and efficient, making it suitable for use in mobile and embedded vision applications. The proposed method uses batch normalization, ReLU activation, and a softmax layer to calculate the confidence probabilities of the input being a traffic sign. The experiments were conducted using the German Traffic Sign Recognition Benchmark (GTSRB) dataset, which consisted of 39,209 training images and 12,630 testing images from 43 classes. The method was trained using the Adam optimizer with a learning rate of 0.001, and the model was trained for 30 epochs without data augmentation and another 200 epochs with data augmentation techniques, such as rotation, scaling, shift, and shear transformations. The final result was an accuracy of 99.66%, demonstrating the effectiveness of the proposed method.

In a recent study, Zhu and Yan (2022) [12] tackled the problem of traffic sign recognition using two deep learning methods: You Only Look Once (YOLO)v5 and the Single Shot MultiBox Detector (SSD). YOLOv5 is a real-time object recognition algorithm that processes the entire image with a single neural network and divides it into parts to estimate the bounding boxes and probabilities for each part. The SSD, on the other hand, accelerates the process by eliminating the need for region proposal networks for each component. The authors collected a dataset of 2182 traffic sign images from 8 different classes, which was split as follows: 64% training set, 16% validation set, and 20% testing set. The models were trained using data augmentation techniques, such as rotation and resizing. For YOLOv5, the image size was set to 640 × 640, the batch size was 16, and the model was trained for 200 epochs. For the SSD, the model was frozen for 100 epochs and then unfrozen and trained for another 200 epochs with a batch size of 16 and an input shape of 300 × 300 × 3. The proposed method achieved an accuracy of 97.70% for YOLOv5 and 90.14% for SSD, demonstrating the effectiveness of the proposed approach in terms of its accuracy.

Shustanov and Yakimov (2017) [13] proposed a Convolutional Neural Network (CNN)-based solution for traffic sign recognition. The proposed CNN architecture includes convolutional, fully connected, and softmax layers. The authors conducted experiments with different configurations of the CNN architecture, with the best one including 3 convolutional layers, 1 fully connected layer, and a softmax layer. The GTSRB dataset, consisting of around 50,000 traffic sign samples, was used for the experiments. The dataset was split as follows: 80% for training and 20% for testing. The proposed method achieved an accuracy of 99.94%.

Alghmgham et al., (2019) [14] developed an autonomous traffic sign detection and recognition system using a deep learning approach with CNN. The proposed CNN architecture consisted of pooling layers, nonlinearity, a dense layer activation function, and a leaky ReLU to address the issue of dead neurons. The softmax function and the categorical cross-entropy function were used to calculate the difference between the output of the softmax function and the real class’s one-hot encoding. The Adam optimizer and Stochastic Gradient Descent were applied in the CNN architecture. The batch size was set to 50, 100, 200, and 400 for 10, 50, 100, and 150 epochs, respectively. The authors self-collected the Arabic Traffic Signs dataset, consisting of 2728 images with 24 classes, such as road humps, right turns, U turns, stops, etc. The images were resized to 30 × 30 pixels, and the dataset was split as follows: 60% for training, 20% for testing, and 20% for validation. The best architecture, with 2 convolutional layers, 2 max pooling layers, and 3 dense layers, achieved an accuracy of 100% with 150 epochs for all batch sizes.

Li et al., (2019) [15] proposed a CNN-based solution for traffic sign recognition. The proposed CNN architecture included 2 convolutional layers, 6 max pooling layers, and 4 traffic sign modules aimed at extracting features from the images. The authors conducted experiments using two datasets, GTSRB and BTSD, with over 50,000 and 7000 images, respectively. The hyperparameters, such as a learning rate of 0.001, gamma set of 0.1, and step values of 24,000 and 48,000 for 60,000 iterations, were set in the experiments. The proposed method achieved an accuracy of 97.4% on the GTSRB dataset and 98.1% on the BTSD dataset.

Yazdan and Varshosaz (2021) [16] presented a novel approach for traffic sign recognition that leverages a minimal set of common images. The proposed method creates a new orthogonal image of the traffic sign and compares it to a database of single images shot in front of each sign, eliminating the need for multiple images in the training database. The orthogonal image is generated from stereo pictures and put through a template-matching procedure. This approach resulted in an accuracy of 93.1% for recognizing traffic signs.

Bangquan and Xiong (2019) [17] proposed a traffic sign recognition system using the Efficient Convolutional Neural Network (ENet), which combines two pretrained models, VGG16, and LeNet. The system was trained on the GTSRB dataset, which comprises 43 classes of traffic signs and was split into a training set of 39,209 and a test set of 12,630. The system was trained using the Adam optimizer with the softmax cross-entropy loss function. The experiment showed that the LeNet model performed better than the VGG16 model, with accuracy levels of 98.6% and 96.7% accuracy, respectively. ENet with the LeNet algorithm was slower and larger but more accurate, while ENet with the VGG16 algorithm was quicker and smaller but less precise. The system demonstrated excellent generalization skills by correctly classifying all images in a new dataset.

Zaibi et al., (2021) [18] proposed an enhanced LeNet-5 model for traffic sign classification. The proposed model consisted of two convolution layers to extract features from images, followed by two stacked convolution layers, and a single fully connected layer. To increase the model’s stability and training speed, batch normalization and dropout with a rate of 0.5 were added after the fully connected layer. The enhanced LeNet-5 was trained on the GTSRB and BTSD datasets, and the images were preprocessed using histogram equalization, grayscale conversion, resizing, and normalization. The experiment showed that the Adam optimizer with ReLU activation performed better than Adadelta with LeakyReLU activation, and that the LeNet-5 model achieved an accuracy of 99.84% on the GTSRB dataset and 98.37% on the BTSD dataset.

In the study conducted by Sreya (2021) [19], a Convolutional Neural Network (CNN) approach was proposed for traffic sign classification. The model architecture consisted of 6 layers, with 4 convolutional layers and 2 max pooling layers, and used the LeNet model as its base. The experiments were performed on the GTSRB dataset with two different batch sizes, 50 and 31,367. Data preprocessing and data augmentation were applied to enhance the size of the dataset and prevent overfitting. The first set of experiments, with a batch size of 50 and 10 epochs, resulted in an accuracy of 66.80%, while the second set of experiments with a batch size of 31,367 and 15 epochs achieved an accuracy of 90.07% at the 12th epoch.

Similarly, Abudhagir and Ashok (2022) [20] leveraged the LeNet model for traffic sign recognition. The first two layers of their CNN architecture were based on the LeNet model, followed by 2 convolutional layers, a dropout layer, and a flatten layer. The GTSRB dataset was used, with 20% of the images used for training and 80% for testing. The dataset was resized and augmented to increase its size. The model was trained for 20 epochs, and achieved an accuracy of 98.50% on the GTSRB dataset.

The study by Mehta et al., (2019) [21] presented a deep Convolutional Neural Network (CNN) for traffic sign classification. The network architecture consists of three convolutional layers followed by two fully connected layers, max pooling layers, and three additional convolutional layers. The input to the network is a color image of size 64 × 64, and the output is a classified RGB image. The authors trained the network with a total of 1 million trainable parameters using the BTSD dataset retrieved from video clips. Optimization was performed with the Stochastic Gradient Descent (SGD) and Adam optimizer with dropout rates of 0.2 and 0.3, and the activation functions used were the sigmoid and softmax functions. The model was trained for 10 epochs, and the testing accuracy achieved was 97.06%.

In contrast, Zhang et al., (2020) [22] proposed a lightweight CNN for traffic sign classification. The study involved two models, a teacher network and a student network. The teacher network was used to train the network, which was then passed on to the student network with fewer layers, to improve the network’s capacity for traffic sign recognition. The architecture of the network involved the use of two 1 × 1 convolutional filters to divide the input feature maps into fewer channels, and then the convolution operations were performed using 1 × 1 and 3 × 3 kernels, which were combined. Six cells were used to create direct links between the various levels of the network, and ReLU and batch normalization were applied in the layers. The student network consisted of five convolutional layers and a fully connected layer with batch normalization and ReLU, and optimization was performed using the Adam optimizer. The network was pruned to reduce the number of training parameters, using techniques such as weight quantization, low-rank, network pruning, and network slimming. The final network had only six layers, and the batch size was set to 128 with a learning rate of 0.001 for 300 epochs. The datasets used for training and testing were the GTSRB and BTSC datasets, and the accuracy obtained from the GTSRB dataset was 99.38%, while that of the BTSC dataset was 98.89%.

In a study by Sokipriala and Orike (2021) [23], several convolutional neural network (CNN) models were evaluated for their performance in traffic sign classification. The three models evaluated were tVGG16, ResNet50, and the authors’ proposed CNN based on AlexNet. The proposed CNN model was designed with reduced filter sizes and a stride size of 1. It also employs Maxpool with a stride size of 2 for feature map downsampling, a flatten layer, and three fully connected layers. In the final fully connected layer, 43 neurons are used to represent each of the 43 different traffic sign classes, along with a softmax activation function for classification. ReLU activation is used after each convolutional layer to prevent the convolved features from averaging to zero. The dataset used was the German Traffic Sign Recognition Benchmark (GTSRB), which consists of 43 traffic sign classes with 34,799 training images, 4410 validation images, and 12,630 testing images. The training set was transformed to grayscale to reduce the intensity and lower the computational cost, followed by histogram equalization for contrast stretching to ensure a uniform distribution of pixel intensities. Data augmentation techniques, such as translation, zoom, rotation, shear, and crop, were applied to address the imbalance in the data. All models were trained using categorical cross-entropy loss, softmax activation, the Adam optimizer, and a learning rate of 0.0001 for 10 epochs in 40 mini batches. The results showed that VGG16 achieved an accuracy of 95.5%, ResNet50 achieved an accuracy of 95.4%, and the proposed CNN with AlexNet achieved the highest accuracy of 96.0%.

Vincent et al., (2020) [24] proposed a traffic sign classification CNN that was evaluated on the GTSRB dataset. The proposed CNN consisted of 4 fully connected layers, 4 convolutional layers, 2 pooling layers, and 1 flatten layer. The images were resized to 32 × 32 pixels, and the kernel size of the first two convolutional layers was 5 × 5. The kernel size of the initial max pooling layer was 2 × 2. The convolutional layer selected the most important patterns, which were accompanied by the max pooling layer. The fully connected layers were designed for categorization, with 256, 128, and 64 hidden nodes in the whole connection layer and 43 hidden nodes in the final output layer. Data preprocessing methods, such as grayscale conversion, histogram equalization, and normalization, were performed to address the poor quality of the images. Data augmentation techniques, such as flip, rotation, and zoom, were also applied to increase the size of the dataset. The hyperparameters were set as a batch size of 128, categorical cross-entropy, the Adam optimizer, ReLU and softmax activation, and dropout rates of 0.2 and 0.5 for 50 epochs. With this method, the proposed CNN achieved an accuracy of 98.44% on the GTSRB dataset.

In Madan et al., (2019) [25], a method was proposed to classify traffic signs using a hybrid combination of HOG features and Speed Up Robust Features (SURF) and a CNN classifier. The study proposed two CNN architectures, the basic CNN architecture and the branching CNN architecture. The basic architecture consists of two convolutional blocks with batch normalization, ReLU activation, and max pooling layers in each block. The features are then passed through the embedding, flattened, and delivered to the fully connected layers. In contrast, the branching CNN architecture has two branches, each consisting of two convolutional blocks. One branch receives HOG features in the form of 7 × 252, and the other branch receives SURF features in the form of 8 × 64. The model applies convolutions to the two distinct feature maps using this branching technique, which constrains resources and lowers model parameters. The experiment was applied to the GTSRB dataset, which contains 39,029 training images, and data augmentation techniques such as brightness, rotation, and distortion with various flips were applied. The basic CNN achieved an accuracy of 98.07%, and the performance significantly improved to 98.48% with the use of the branching CNN architecture.

In Serna and Ruichek (2018) [26], several CNN models were used to perform traffic sign classification. The datasets used were GTSRB and the European Traffic Sign Dataset (ETSD), which was self-collected by the authors and consists of traffic signs from six European countries. The ETSD was split into four main categories (warning signs, regulatory signs, informative signs, and others) with a total of 82,476 images and 164 classes split as follows: 60% training set, 30% testing set, and 10% validation set. Data augmentation techniques, such as the width and height shift, scaling, shear, and rotation, were applied in the study. The models used were LeNet-5, IDSIA, URV, CNN asymmetricK, and 8-layer CNN. The architecture of the CNN asymmetricK consisted of 3 symmetric convolutions, 6 asymmetric convolutional layers, 2 fully linked layers, and batch normalization and ReLU activations. A preprocessing stage was also applied to convert the images to the HSV color scheme and equalize the V channel. The 8-layer CNN consisted of convolutional layers triggered by the ReLU function, followed by max pooling and dropout layers, with L2 regularization applied before the ReLU activations on each fully connected and convolutional layer. The results show that the CNN asymmetricK and 8-layer CNN achieved almost the same accuracy for both datasets, but the 8-layer CNN achieved a slightly higher accuracy with an accuracy of 99.37% for GTSRB and 98.99% for ETSD.

Mishra and Goyal (2022) [27] proposed a traffic sign classification and recognition method with the deep CNN. In the architecture of the proposed CNN model, there are convolutional layers, a pooling layer, and a max pooling layer with a range of dropouts. Three datasets were used for the experiment, including GTSRB, BTSC, and TSRD+GTSRB, with 43, 62, and 101 classes, respectively. In order to avoid overfitting and to increase the generality, data augmentation techniques, such as rotation, zoom, and rescale were applied. All datasets were split as follows: 70% training images and 30% testing images. With the proposed CNN method, GTSRB achieved an accuracy of 99.76%, BTSC achieved an accuracy of 99.79%, and TSRD+GTSRB achieved an accuracy of 98.37%.

In a study by Chen et al., (2017) [28], a two-CNN approach was used for traffic sign classification. The two CNNs were the Combined CNN (CCNN) and the Multicategory CNN (MCNN). The MCNN model was trained using the original dataset, while the CCNN model was trained with data-augmented samples. The results of both models were compared by computing the probabilities of the superclass and subclass of the traffic signs and choosing the actual recognition label based on the higher probability weight. The dataset used was the GTSRB dataset with 43 classes and consisted of 26,640 training images and 12,569 testing images. The images were resized to 32 × 32 and underwent data augmentation techniques, such as scaling, rotation, and flipping, to increase the sample size of the dataset. The 43 subclasses in the GTSRB dataset were grouped into five superclasses, including red circular prohibitory signs, red triangular danger signs, blue circular mandatory signs, black circular derestriction signs, and other signs. The experiments were run for 100 epochs with a batch size of 30. The results showed that the CCNN with data augmentation achieved a higher accuracy level (98.26%) compared to the MCNN model (97.96%).

Zheng and Jiang (2022) [29] carried out a comprehensive evaluation of different CNN models and Vision Transformer (ViT) models for traffic sign classification. The CNN models evaluated included VGG16, ResNet, DenseNet, MobileNet, SqueezeNet, ShuffleNet, and MnasNet. The ViT models evaluated were RealFormer, Sinkhorn Transformer, Nyströmformer, and Transformer in Transformer (TNT). The experiments were conducted on three datasets: the German Traffic Sign Recognition Benchmark (GTSRB) with 43 classes, the Indian Cautionary Traffic Sign (ICTS) with 15 classes, and the CSUST Chinese Traffic Sign Detection Benchmark (CCTSDB) with 103 classes. The experiments used a batch size of 64, the Adam optimizer, a learning rate of $3\times 10^{-5}$, and a cross-entropy loss function. The CNN models were trained for 20 epochs on all three datasets, while the ViT models were trained for 50 epochs on the GTSRB and Chinese datasets and 100 epochs on the Indian dataset.The CNN models were trained for 20 epochs on all three datasets, while the ViT models were trained for 50 epochs on GTSRB and the Chinese dataset and 100 epochs on the Indian dataset. The results showed that the DenseNet model achieved an accuracy of 98.82% on GTSRB, ShuffleNet achieved an accuracy of 99.11% on the Indian dataset, and DenseNet achieved an accuracy of 99.42% on the CCTSDB dataset. On the other hand, the RealFormer ViT model achieved an accuracy of 86.03% on GTSRB, and the TNT ViT model achieved an accuracy of 95.05% on the CCTSDB dataset. The highest accuracy achieved by a ViT model was 97.10% on the ICTS dataset, which was obtained without the use of any ViT models. Overall, the experimental results indicated that CNN models are more effective than the ViT models for traffic sign classification.

Haque et al., (2021) [30] introduced a lightweight CNN architecture called DeepThin, which stacks several convolutional layers with modest kernel sizes. The authors found that they performed the best with 32 and 48 filter channels with all convolutional layers having a kernel size of 3 × 3. The loss function used was cross-entropy, and the optimizer was the Stochastic Gradient Descent (SGD). The datasets used were the GTSRB and the Belgian Traffic Sign Classification (BTSC) dataset with the images resized to 45 × 45 pixels and converted to grayscale. DeepThin achieved an accuracy of 99.72% for GTSRB and 99.29% for BTSC after incorporating techniques such as ensemble learning and fine-tuning. The authors observed that the RGB dataset performed better than grayscale images.

Usha et al., (2021) [31] presented another CNN model for traffic sign classification consisting of convolutional, pooling, and dropout layers. The activation function used was ReLU and the categorical cross-entropy loss function was optimized using the Adam optimizer. The model was trained on the GTSRB dataset and achieved an accuracy of 97.8% after only 15 epochs.

Fang et al., (2022) [32] presented a method for traffic sign classification using MicronNet-BN-Factorization (MicronNet-BF). MicronNet is a small deep neural network designed for use in embedded devices, and MicronNet-BF improved its accuracy by integrating it with batch normalization and factorization. The dataset used was the GTSRB dataset with 43 classes, and MicronNet-BF achieved an accuracy of 99.38% with a processing time of only 1.41 s. The inclusion of batch normalization improved the accuracy by 1.05% compared to the original MicronNet.

Sarku et al., (2021) [33] introduced a novel approach to traffic sign recognition by leveraging several Residual Neural Networks (ResNets), including ResNet18, ResNet50, and ResNet152. These ResNets had 18, 50, and 152 weighted hidden layers, respectively. The authors collected a dataset of over 40,000 images using a self-driving car, but only used 224 high-resolution images for their experiments. The dataset consisted of three classes, stop, do not enter, and crosswalk, each containing 300 images. The images were resized to 224 × 224 pixels and split as follows: 80% training, 20% validation. Fifteen images were used for testing. No data augmentation was applied to the dataset. The model was trained for 10 epochs with a batch size of 10, and the last fully connected layer was fine-tuned. The highest test accuracy was 93% for ResNet50, followed by 60% for ResNet18 and 33% for ResNet152.

Cao et al., (2019) [34] proposed an improved LeNet-5 CNN architecture for traffic sign classification. The LeNet-5 architecture included 2 convolutional layers, 2 pooling layers, 2 fully connected layers, and a classification layer. To improve the model’s performance, a Gabor kernel was used as the first convolutional kernel, and batch normalization was introduced after each pooling layer. The ReLU activation function was used to address gradient vanishing and exploding issues, and a dropout rate of 0.5 was applied in the fully connected layers. The authors used the GTSRB dataset with 75% used for training and 25% for testing. The LeNet-5 model achieved an accuracy of 99.75% on the GTSRB dataset.

Fu and Wang (2021) [35] proposed a multiscale convolutional network (MSCN) and a multicolumn deep neural network (MCDNN) approach to traffic sign recognition. The TSRD dataset was used for training, while the GTSRB dataset was used for testing. The authors performed data augmentation and fine-tuning to improve the model’s performance. However, the results showed a tendency for the accuracy to increase initially and then decline as the number of classes increased. The proposed method achieved an accuracy of 90.13%.

In Sichkar and Kolyubin (2019) [36], a study was conducted to explore the impact of different dimensions of convolutional layer filters on the performance of a CNN for traffic sign classification. The dimensions considered in the experiment were 3, 5, 9, 13, 15, 19, 23, 25, and 31. The authors used the GTSRB dataset for their experiments. The images in the dataset were preprocessed by normalizing them and resizing them to 32 × 32 pixels. The negative log-likelihood loss function was employed for network optimization, and 1 stride was used for the convolutional layer and 2 strides for the pooling layer. The results showed that the convolutional layer filters with 9 × 9 and 19 × 19 dimensions yielded the highest accuracy levels of 86.4% and 86.8%, respectively, with fast classification speeds of 0.004472 and 0.002786 s.

Agarwal et al., (2022) [37] proposed a CNN-based method for traffic sign classification. The proposed CNN architecture consists of 12 layers, including 4 convolutional layers, 2 max pooling layers, 4 dropout layers, 1 flatten layer, and 1 fully connected layer. The categorical cross-entropy loss function was used to optimize the network, and the experiments were conducted on the GTSRB dataset. The images were resized to 30 × 30, and a batch size of 30 was used. The dataset was split as follows: 75% for training and validation and 25% for testing. The proposed method achieved an accuracy of 99.66%.

Similarly, Youssouf (2022) [38] leveraged a CNN for traffic sign classification. The model consisted of 4 convolutional layers, 2 max pooling layers, a dropout layer, a flatten layer, and 2 dense layers. A convolutional filter size of 3 × 3 was applied in the architecture, and the ReLU activation function was employed in various hidden layers. The categorical cross-entropy loss function, the Adam optimizer, and a learning rate of 0.001 were also used. The GTSRB dataset was partitioned as follows: 60% for training, 20% for validation, and 20% for testing. Data augmentation techniques, such as random spinning, stretching, and flipping, were applied to balance the class sample distributions and reduce bias. The proposed CNN architecture achieved an accuracy of 99.20% with a classification speed of 6.63 s for the testing data.

G"okberk et al., (2022) [39] compared three CNN models: AlexNet, DarkNet-53, and EfficientNet-b0. The AlexNet architecture consisted of 13 layers including 5 convolutional layers, 3 max pooling layers, 2 dropout layers, and 3 fully connected layers. The activation functions used were ReLU and Softmax. The DarkNet-53 architecture was made up of 53 layers, mostly 1 × 1 and 3 × 3 convolutional layers, and a batch normalization layer and a LeakyReLU layer after each convolutional layer. EfficientNet-b0 consisted of 7 MBConv layers, a convolutional layer, and a pooling and fully connected layer. The GTSRB dataset was used for the experiment, which was split as follows: 70% training data, 15% validation data, and 15% testing data. The input shape for each algorithm was different, with AlexNet being 227 × 227, DarkNet-53 being 416 × 416, and EfficientNet-b0 being 224 × 224. The results showed that EfficientNet-b0 achieved the highest accuracy of 98.64%, followed by AlexNet with 97.45% and DarkNet-53 with 94.69%.

Kuros and Kryjak (2022) [40] proposed a traffic sign classification method using both the Deep Neural Network (DNN) and Quantum Neural Network (QNN). They used a collection of *N* quantum filters to build quantum convolution (quanvolution) layers. Both the DNN and QNN had layers with convolutional, max pooling, dropout, flatten, and dense properties. The images were convolved using quantum circuits, and the GTSRB dataset was used, which was split as follows: 80% training set, 10% test set, and 10% validation set. The results showed that the DNN achieved an accuracy of 99.86%, while the QNN obtained an accuracy of 94.40%.

Pradana et al., (2022) [41] also proposed a traffic sign classification method using the CNN. Their network consists of 3 fully connected layers, 3 max pooling layers, and 3 convolution layers. The network was designed to convert a 100 × 100 grayscale image into a smaller size and classify it into one of 41 traffic sign classes. ReLU was used as the activation function and the network was trained for 10 epochs. The Indonesian traffic sign dataset was used, which included 3133 traffic sign pictures and 41 classifications. The proposed architecture achieved an accuracy of 93%.

Bhatt et al., (2022) [42] proposed a traffic sign classification model using CNNs. The model is made up of 11 layers, including four convolutional layers, two pooling layers, one flattening layer, and four fully connected layers. The model was trained using the GTSRB dataset and a self-collected Indian traffic sign dataset. A hybrid dataset was formed by combining the two datasets, resulting in 102 classes in total and 65,810 images. Preprocessing steps, such as grayscaling, histogram equalization, and normalization, were applied to the dataset. The model achieved accuracy levels of 99.85% on the German dataset, 91.08% on the Indian dataset, and 95.45% on the hybrid dataset.

Mamatkulovich (2022) [43] proposed a lightweight CNN model for traffic sign classification. The architecture of the model consists of six layers, including four convolutional layers, a fully connected layer, and a classification layer. The first four layers were designed as residual blocks to enhance the model’s performance. ReLU was used as the activation function, and a dropout rate of 0.2 was applied as a regularization step. The model was trained using the GTSRB dataset, which was resized to 32 × 32 pixels and normalized. The model achieved an impressive accuracy of 99.93%. Table 3 provides a summary of the deep learning methods for traffic sign recognition.

## 5. Traffic Sign Recognition Datasets

There are several datasets in the field of traffic sign recognition that are commonly used to evaluate the performance of recognition algorithms. These datasets provide a comprehensive and diverse representation of real-world scenarios and traffic signs, allowing researchers to train and test their algorithms in realistic environments.

### 5.1. German Traffic Sign Recognition Benchmark (GTSRB)

The German Traffic Sign Recognition Benchmark (GTSRB) [44] is a well-established and widely utilized dataset in the field of traffic sign recognition. With a total of 51,839 high-resolution images covering 43 unique traffic sign classes, the GTSRB provides a comprehensive and reliable resource for evaluating the performance of traffic sign recognition algorithms. However, it is important to note that the GTSRB dataset primarily consists of German traffic signs, which may not accurately represent the diversity of traffic signs used in other regions. This limits the generalization of models trained on the GTSRB dataset and may result in a decreased performance when applied to other regions. Despite this limitation, the GTSRB dataset remains a popular resource due to its size, high-quality annotations, and real-world scenario representation, making it an excellent resource for researchers in the field of traffic sign recognition.

### 5.2. Belgium Traffic Sign Dataset (BTSD)

The Belgium Traffic Sign Dataset (BTSD) [45] is a widely acknowledged dataset in the field of traffic sign recognition, comprising over 7095 high-resolution images, representing 62 unique traffic sign classes found in both Belgium and the Netherlands. The images were carefully collected from a range of real-world scenarios, providing a diverse representation of lighting conditions, weather, and occlusions. While the BTSD dataset is relatively small compared to other datasets in the field, it still provides a valuable resource for researchers, particularly for validating the performance of models trained on larger datasets, such as GTSRB or JTSRB. The BTSD is recognized for its high-quality images and diverse representation of real-world conditions, making it a useful resource for researchers working on developing traffic sign recognition systems specifically for Belgium and the Netherlands.

### 5.3. Chinese Traffic Sign Database (TSRD)

The Chinese Traffic Sign Database (TSRD), funded by the National Nature Science Foundation of China (NSFC), is a comprehensive database of traffic sign images. With a total of 6164 images divided into 58 different traffic sign classes, the TSRD provides a rich and realistic dataset for training and evaluating traffic sign recognition algorithms. The images in the TSRD were collected from various sources, including BAIDU Street View and cameras placed in real-world settings, resulting in a diverse range of images captured under varying conditions, such as different weather conditions, lighting conditions, and surroundings. The database also includes images depicting partially occluded signs, providing a challenging scenario for testing the robustness of recognition algorithms and simulating real-world conditions. Overall, the TSRD is a valuable resource for researchers working on traffic sign recognition due to its diverse and realistic representation of traffic signs.

All of these datasets have unique strengths and limitations, and the choice of which dataset to use will depend on the specific needs and requirements of the recognition system being developed. Factors such as the size of the dataset, the variety of traffic sign classes represented, and the quality of the images all need to be considered when selecting the most appropriate dataset for a particular use case.

A summary of the traffic sign recognition datasets is provided in Table 4.

## 6. Limitations

Traffic sign recognition is a crucial task in the field of computer vision and autonomous driving; however, there are still several limitations to the existing works in this field. Some of the most significant limitations are as follows:

- Illumination Variations: Traffic signs may be underexposed, overexposed, or partially illuminated due to changing lighting conditions, which can affect the accuracy of traffic sign recognition systems. One way to address this issue is to use advanced image processing techniques, such as histogram equalization, contrast stretching, or color space transformations, to enhance the visibility of the sign. Another method is to use multiple cameras and sensors to capture different views of the sign and combine them to form a more accurate representation.
- Occlusions: Traffic sign recognition systems may also fail to recognize signs that are partially or fully occluded by other objects such as trees, buildings, or other vehicles. One solution to this problem is to use contextual information, such as the road layout, vehicle speed, and direction of travel, to predict the presence of a sign. Another approach is to use deep learning techniques, such as object detection or semantic segmentation, to identify and separate the sign from the surrounding objects.
- Sign Damage or Degradation: Over time, traffic signs may become damaged, faded, or covered in dirt, which can make them difficult to recognize. To address this issue, traffic sign recognition systems can be trained on a diverse range of sign images, including those that are degraded, to improve their robustness to signs with varying conditions. Another approach is to use active learning to continually update the model with new examples and retrain the system to adapt to changes in the environment.
- Limited Training Data: One of the main challenges of traffic sign recognition systems is the availability of labeled training data. To overcome this limitation, synthetic data generation methods can be used to generate artificial images that simulate real-world conditions. Additionally, transfer learning can be used to fine-tune pretrained models on smaller datasets to achieve a better performance.

Overall, the field of traffic sign recognition is still in its early stages, and there is significant room for improvement and further research. Addressing these limitations will be critical for developing algorithms that can perform well in real-world conditions and contribute to the development of safer and more efficient autonomous systems.

## 7. Future Research Prospects

The field of traffic sign recognition continues to evolve, and there are several promising avenues for future research. Some of the key areas of focus for future research in traffic sign recognition include the following:

- Deep learning: With the recent advancements in deep learning techniques, there is potential for significant improvements in the performance of traffic sign recognition algorithms. Researchers are exploring the use of deep neural networks to improve the accuracy and robustness of recognition systems, particularly in challenging conditions, such as at night-time and in adverse weather.
- Real-time processing: Real-time processing is critical for practical applications of traffic sign recognition, particularly for autonomous vehicles. Researchers are exploring the use of specialized hardware, such as graphics processing units (GPUs), to accelerate the processing time of recognition algorithms, as well as developing algorithms that can run on resource-constrained devices.
- Cross-dataset generalization: One of the current limitations of existing traffic sign recognition algorithms is their lack of generalization across different datasets. Future research will aim to address this issue by developing algorithms that can learn from multiple datasets and be generalized to new, unseen data.
- Robustness to variations: Traffic signs can vary greatly in appearance, even within the same class. Future research will aim to improve the robustness of recognition algorithms to variations in sign appearance, such as changes in color, texture, or orientation, by incorporating domain-specific knowledge or developing novel deep learning models.

Overall, the future of traffic sign recognition is promising with significant opportunities for research and development. These efforts will ultimately lead to more accurate and reliable recognition systems, enabling a safer and more efficient transportation infrastructure.

## 8. Conclusions

In conclusion, the recognition of traffic signs is a crucial aspect of autonomous vehicle technology, as it enables vehicles to drive safely and efficiently. The recent advancements in computer vision and machine learning have greatly improved the accuracy of traffic sign recognition. This paper provides a comprehensive overview of the various techniques and approaches used in traffic sign recognition, including preprocessing, feature extraction, classification, datasets, and performance evaluation. The paper also highlights the commonly used traffic sign recognition datasets and the challenges associated with them. Despite the progress made in this field, the variability of traffic signs across different regions, complex background scenes, and changes in illumination remain significant challenges that need to be addressed. This paper provides insights into the limitations and future research prospects in traffic sign recognition and will serve as a useful resource for researchers and practitioners in the field of autonomous driving and traffic sign recognition.

## Figures and Tables

**Table 1:** Comparison of Machine Learning and Deep Learning Approaches for Traffic Sign Recognition.

Aspect	Machine Learning	Deep Learning
Architecture	Use a simpler architecture and handcrafted features as input to a classifier, such as decision tree or support vector machine.	Use neural networks with many layers to automatically learn relevant features from the input data.
Performance	Achieve a lower accuracy level than deep learning models on large and complex datasets.	Achieve a higher accuracy level than machine learning models on large and complex datasets.
Data requirements	Perform well on smaller datasets and are less susceptible to overfitting.	Generally require large amounts of data to achieve a high accuracy level.
Training time	Train faster than deep learning models, especially when training on smaller datasets.	Can take longer to train than machine learning models, especially when training on large datasets.
Interpretability	Generally more interpretable than deep learning models, often using simple and transparent classifiers.	Can have many layers that make it difficult to understand how the model is making its predictions.
Transfer learning	Less effective at transfer learning as handcrafted features may not generalize well to new datasets.	Well-suited for transfer learning, where a pretrained model can be fine-tuned on a new dataset with relatively few examples.

**Table 2:** Summary of Machine Learning Approaches to Traffic Sign Recognition.

Author	Algorithm	Dataset	Accuracy (%)
Kerim and Efe (2021) [1]	ANN	GTSRB	95
Soni et al., (2019) [2]	LBP, HOG, PCA, SVM	TSRD (Chinese)	84.44
Namyang and Phimoltares (2020) [3]	HOG, CLD, SVM, Random Forest	Self-collected (Thai)	93.98
Li et al., (2022) [4]	Color Histogram, HOG, PCA	GTSRB	99.99
Madani and Yusof (2018) [5]	Border Color, Shape, Pictogram, SVM	GTSRB	98.23
Sapijaszko et al., (2019) [6]	DWT, DCT, MLP	BTSD	96.0
		GTSRB	95.7
		TSRD	94.9
Aziz and Youssef (2018) [7]	HOG, CLBP, Gabor, ELM	GTSRB	99.10
		BTSC	98.30
Weng and Chiu (2018) [8]	CCL, HOG, SVM	GTSRB	90.85
Wang (2022) [9]	LR	GTSRB	97.75
	MLP		98.88
	SVM		95.51

**Table 3:** Summary of Deep Learning Approaches to Traffic Sign Recognition.

Author	Algorithm	Dataset	Accuracy (%)
Siniosoglou (2021) [10]	Deep Autoencoder	CATERED	99.19
Li and Wang (2018) [11]	MobileNet	GTSRB	99.66
Zhu and Yan (2022) [12]	YOLOv5	Self-collected	97.70
Shustanov and Yakimov (2017) [13]	CNN	GTSRB	99.94
Alghmgham et al., (2019) [14]	CNN	Self-collected (Arabic)	100
Li et al., (2019) [15]	CNN	GTSRB	97.4
		BTSD	98.1
Yazdan and Varshosaz (2021) [16]	Normalized cross-correlation (NCC)	BTSD	93.10
Bangquan and Xiong (2019) [17]	LeNet	GTSRB	98.6
	VGG16		96.7
Zaibi et al., (2021) [18]	LeNet-5	GTSRB	99.84
		BTSD	98.37
Sreya (2021) [19]	CNN (LeNet)	GTRSB	90.07
Abudhagir and Ashok (2022) [20]	CNN (LeNet)	GTRSB	98.50
Mehta et al., (2019) [21]	CNN	BTSD	97.06
Zhang et al., (2020) [22]	CNN	GTSRB	99.38
		BTSC	98.89
Sokipriala and Orike (2021) [23]	ResNet50	GTSRB	95.4
	VGG16		95.5
	CNN		96.0
Vincent et al., (2020) [24]	CNN	GTSRB	98.44
Madan et al., (2019) [25]	Basic CNN	GTSRB	98.07
	Branching CNN		98.48
Serna and Ruichek (2018) [26]	CNN	GTSRB	99.37
		ETSD	98.99
Mishra and Goyal (2022) [27]	CNN	GTSRB	99.76
		BTSC	99.79
		TSRD + GTSRB	98.37
Chen e t al. (2017) [28]	MCNN	GTSRB	97.96
	MCNN		98.26
Zheng and Jiang (2022) [29]	DenseNet	GTSRB	98.82
		CCTSDB	99.42
	ShuffleNet	ICTS	99.11
	RealFormer	GTSRB	86.03
	TNT	CCTSDB	95.05
Haque et al., (2021) [30]	DeepThin	GTSRB	99.72
		BTSC	99.29
Siniosoglou (2021) [10]	Deep Autoencoder	CATERED	99.19
Usha et al., (2021) [31]	CNN	GTSRB	97.80
Fang et al., (2022) [32]	MicronNet-BF	GTSRB	99.38
Sarku et al., (2021) [33]	ResNet18	Self-collected	60
	ResNet50		93
	ResNet152		33
Cao et al., (2019) [34]	LeNet-5	GTSRB	99.75
Fu and Wang (2021) [35]	MSCN + MCDNN	TSRD (train), GTSRB (test)	90.13
Sichkar and Kolyubin (2019) [36]	CNN	GTSRB	86.80
Agarwal et al., (2022) [37]	CNN	GTSRB	99.66
Youssouf (2022) [38]	CNN	GTSRB	99.20
Gökberk et al. (2022) [39]	AlexNet	GTSRB	97.45
	DarkNet-53		94.69
	EfficientNet-b0		98.64
Kuros and Kryjak (2022) [40]	DNN	GTSRB	99.86
	QNN		94.40
Pradana et al., (2022) [41]	CNN	Indonesian Traffic Signs	93.00
Bhatt et al., (2022) [42]	CNN	GTSRB	99.85
		Self-collected (Indian),	91.08
		GTSRB+Indian	95.45
Mamatkulovich (2022) [43]	CNN	GTSRB	99.93

**Table 4:** Summary of GTSRB, BTSD, and TSRD.

Dataset	Classes	Total Samples
GTSRB	43	51,839
BTSD	62	7095
TSRD	58	6164

## Data Availability

Data sharing not applicable.
